# Needs and Preferences of Middle-Aged and Older Adults in Taiwan for Companion Robots and Pets: Survey Study

**DOI:** 10.2196/23471

**Published:** 2021-06-11

**Authors:** Ching-Ju Chiu, Shiuan Hsieh, Chia-Wei Li

**Affiliations:** 1 Institute of Gerontology, College of Medicine National Cheng Kung University Tainan Taiwan

**Keywords:** middle-aged adults, older adults, companionship demand, robot, pet, acceptance

## Abstract

**Background:**

In recent years, robots have been considered a new tech industry that can be used to solve the shortage in human resources in the field of health care. Also, animal-assisted therapy has been used to provide assistance, companionship, and interaction among the elderly and has been shown to have a positive impact on their emotional and psychological well-being. Both pets and robots can provide dynamic communication and positive interaction patterns. However, preferences for middle-aged and older adults in this regard are not clear.

**Objective:**

This study explored the degree of acceptance of robots and pets as partners in later life and to determine the needs and preferences of elderly individuals related to companion robots.

**Methods:**

A total of 273 middle-aged and older adults aged ≥45 years and living in the community were invited to answer a structured questionnaire after watching a companion robot video. Sociodemographic data, physical health status and activities, experience with technology, eHealth literacy, and acceptance and attitude toward robots and pets were recorded and analyzed using multinomial logistic regression analysis.

**Results:**

Age, level of education, type of dwelling, occupation, retirement status, number of comorbidities, experience with pets, experience using apps, and eHealth literacy were significantly associated with acceptance of robots and pets. Middle-aged and older women preferred robots with an animal-like appearance, while men preferred robots that resembled a human adult. In terms of robot functions, participants preferred a companion robot with dancing, singing, storytelling, or news-reporting functions. Participants’ marital status and whether or not they lived alone affected their preference of functions in the companion robot.

**Conclusions:**

Findings from this study inform the development of social robots with regard to their appearance and functions to address loneliness in later life in fast-aging societies.

## Introduction

Along with increases in the size of the aging population, the demands for care and medical and health care manpower for the elderly population are also increasing. Determining how to adapt to these changes, using limited resources to meet the needs of care recipients, and reducing the burden on caregivers so that middle-aged and older adults have a high quality of life in their old age is an important issue that cannot be ignored.

Cowan [1] divided the issues to be faced by an aging society into 8 categories: dependent living, fall risk, chronic disease, dementia, social isolation, depression, poor well-being, and poor medication management. However, an existing literature review pointed out that in order to establish relevant advanced-age health technologies designed to solve the issues mentioned above, the issues could be divided into 6 groups: general information and communications technology (ICT), robotics, telemedicine, sensor technology, medication management applications, and video games [1].

Over the past decade, the elderly population has been the demographic with the fastest growing use of technological products such as the internet and computers [2,3], and a growing number of studies have shown that health-related ICTs can effectively reduce medical expenditures and care costs and enhance the quality of life of middle-aged and older adults [4,5]. In addition, technological products can help middle-aged and older adults live independently at home and provide health care and medical services in remote areas through mobile health (mHealth) strategies [6]; among these technological products are robots that can assist humans in performing repetitive and dangerous work and become the additional manpower needed for health care [7].

A health care robot is a robot that monitors or promotes physical and mental health and mitigates social psychological problems in the elderly. According to their functions, these robots can be divided into 2 types: rehabilitation robots and social robots [8]. Rehabilitation robots are auxiliary devices that provide physical assistance and make it easier for users to perform physical tasks. They include such things as smart wheelchairs, artificial limbs, and exoskeletons. Social robots interact with the elderly, providing companionship or improving daily life. These robots can be further divided into service-type robots and companionship robots. The function of the service-type robot is mainly related to supporting the independent life of the elderly individual, such as assisting with eating, bathing, toileting, or dressing, as well as performing housework and providing health and safety monitoring. A companionship robot promotes the physical and mental health of elderly persons and enhances their quality of life through companionship, such as the robotic seal PARO that accompanies elderly individuals with dementia; the robot Huggable, which was specially developed for elderly care experimental research; and the robotic dog Aibo, which was intended to improve the quality of life of older individuals and disabled patients [9,10]. Studies have shown that older people prefer less human-looking robots [11,12] and especially enjoy pet-like robots, which are widely used in the care of elderly persons with intellectual disabilities and provide pet-like companionship in lieu of real animals [13,14]. For example, the therapeutic robotic seal pet PARO, which was developed in Japan in 2004, has a body covered with more than 100 sensors and can interact with people. Survey results show that because PARO’s appearance is unfamiliar to people, it is less likely that people will feel a sense of artificial interaction with an animal, and it is more likely to be accepted by the elderly [15]. Many studies have also shown that PARO can improve depression, increase social interaction, and positively stimulate cognitive functions in elderly persons with dementia [16,17], which suggests that robot-assisted therapy is a new therapeutic tool for use among the elderly [18-20]. According to the literature review [21], robot-assisted therapy is beneficial to the moods and behavior of elderly persons. A pet-like social robot can stimulate elderly persons to interact and talk with others and remind older adults of their past experiences with companion pets, while posing fewer concerns about safety (such as attacks or bites) and hygiene (allergies, infections, or dirt) that are associated with real pets. Older adults with dementia can also get the same emotional comfort from robot-assisted therapy as they would from their interaction with real pets. As a result, a pet-like robot provides not only simple entertainment but also assistance, companionship, therapy, interaction, and stimulation, as well as other functions and services [21].

However, technology may not be a substitute for human assistance, companionship, and interaction. A study was conducted to enable “robotic dog doctors” to accompany the elderly through animal-assisted therapy, and the results of the study showed that it had a positive effect on the mental and social health of the elderly participants. The study indicated that animal-assisted therapy can improve emotional and behavioral problems, as well as problems with aggression, in elderly individuals with dementia and can have a positive effect on the mental and social health of all elderly persons. Animal-assisted therapy is often recommended as a goal-oriented nonpharmaceutical therapy for mental problems [22]. For example, a study by Garrity et al [23] on widowed and socially isolated elderly persons over the past year found that those who had no experience with keeping pets were more depressed than those who had such experience. There are also studies showing that keeping pets is related to the survival rate of cardiovascular disease in the elderly [24], suggesting that pet companionship has a curative effect that cannot be ignored in clinical care and treatment. Some scholars have suggested that patients with dementia can experience a reduction in their degree of loneliness and engage in social interaction by interacting with robot pets and get pleasure and attention from it as well as spiritual comfort [25,26]. Thus, robot pets provide a new therapeutic option for the elderly with dementia. Furthermore, animal protection regulations in countries in addition to a lack of adequate animal training makes robot pets more attractive than animal pets. For example, people generally have doubts about the safety and health of animals in Taiwan, which leads to a lot of restrictions on their implementation in therapy [27]. Therefore, robots or robot pets provide the elderly with dynamic, 2-way communication and a positive interaction mode, which can be regarded as another option for them in later life. Robots can do more dangerous and tiring work in the home care of elderly persons, but they may undergo failure and present financial and ethical issues. Although pets have more spontaneous reactions and richer emotional responses and can provide more tactile stimulation, they present safety and health issues that must be taken into consideration, as well as extra time-consuming care requirements. The aforementioned factors affect the user’s choice. According to the theory of planned behavior proposed by Ajzen [28], the occurrence of a behavior depends on the intensity of people’s intentions, and the intensity of intentions is determined by 3 factors: attitude, subjective norms, and perceived behavioral control. Therefore, it is important to explore what factors affect the acceptance of robots and pets among middle-aged and older adults in Taiwan and to understand whether these factors correspond to the theory of planned behavior.

Thodberg et al [29] performed a study that compared pets with robotic dogs. At the beginning of the study, the robotic pet PARO and real pets (dogs) had the same impact on residents. However, with increases in interaction time, residents decreased their conversation and eye contact with PARO, but their focus on and interaction with the dogs remained stable [29]. The study also found that the real animals had more spontaneous and richer emotional responses than the robotic pets and that subjects could get more active tactile stimulation. Compared with toy animals, both robotic pets and real animals can provide 2-way dynamic communication, so it is feasible to use a robot/robot pet as a companion object and an auxiliary technological device for stimulating the sensory and cognitive functions of elderly individuals. However, most of the existing studies exploring the effectiveness of robot pet interventions in the elderly population (eg, psychological and behavioral effects and impact on quality of life) have been conducted on institutionalized elderly individuals with dementia for which long-term care was provided [30-33]; few of the studies have included elderly persons in the community as the study population. In particular, there has been a lack of study on the attitudes, degree of acceptance, and needs of middle-aged and older adults as they relate to robots. In the past, there have also been no studies comparing pets with robots in terms of their use as companion objects. This study is aimed toward closing these gaps in the existing literature to discuss the companion needs of middle-aged and older adults in Taiwan in order to understand their choices of robots or live pets as companion objects in later life and to further discuss the preferences of middle-aged and older adults for companionship robots, as well as other related factors.

## Methods

### Participants

Adults over age 45 years in Taiwan were invited to participate in this study using a convenience sampling method based on the sample selection standard. The number of participants needed for the study was determined as the number of variables (n=26) multiplied by 10. Thus, a total of 273 older adults living in the community comprised the sample. The questionnaire was distributed in gathering places for the elderly in Taiwan such as community universities, senior citizen learning centers, community care strongholds, day-care strongholds, and hospital clinics all over Taiwan. The inclusion criteria for participants were as follows: (1) able to communicate in Mandarin and Taiwanese; (2) willing to be interviewed by researchers, to fill out the questionnaire on their own, or to fill out an electronic questionnaire with a tablet computer; and (3) agreed to participate in the study and to sign a consent form. The exclusion conditions were as follows: (1) resided somewhere other than Taiwan, and (2) were suffering from moderate to severe cognitive impairment or unable to answer questions without coercion.

### Measures

A structural questionnaire was used as the research tool. The content of the questionnaire included 4 parts: sociodemographic data, physical and mental health status and activities, technology use and eHealth literacy, and robot and pet experience.

The sociodemographic data included age, gender, education level, marital status, number of children, place of residence, whether living alone or not, working status, economic status, self-rated health status, and number of chronic diseases.

The physical and mental health status and activities included social participation, leisure activities, social support, depression status, and personality traits. Among them, social participation and leisure activities were measured using the Ministry of Health and Welfare Taiwan Longitudinal Study on Aging questionnaire [34]. Social support was measured using the Inventory of Socially Supportive Behavior (ISSB [35,36]). This inventory consists of 10 questions relating to 4 types of social support: emotional support (3 questions), information support (2 questions), substantive support (2 questions), and social integration (3 questions). In the scoring system for the ISSB, a score of 1 represents unsatisfied, 2 represents neutral, and 3 represents satisfied; the higher the score, the higher the perceived social support. An overall internal consistency coefficient of .91 represents emotional support for each support type α reliability coefficient; a coefficient of .81 represents information support; a coefficient of .73 represents substantive support; and a coefficient of .81 represents social integration [37]. The depression status was measured using the simplified, 10-item version of the Center for Epidemiologic Studies Depression Scale (CES-D [38]), which was translated into Chinese. The CES-D comprises 10 positive and 10 negative questions that are scored on Likert scales ranging from 0 to 3, with the total score ranging from 0 to 30 and a total score of greater than 10 representing depression. The overall internal consistency Cronbach α value ranges from .78 to .87 [39]. The personality assessment was carried out using the International Personality Item Pool (IPIP) big 5 personality scale developed by Goldberg in 1992 [40], which was first translated into a simplified Chinese version [41] and then changed to a traditional Chinese version with customary modifiers used by the Taiwanese population [42]. A single factor was screened out from the original 50 questions, and the questions with higher factor loadings in each domain were developed into a new, 15-item version of the IPIP (IPIP-15). This simplified version of the IPIP big 5 personality scale is divided into 5 dimensions—extroversion, friendliness, rigorousness, emotional stability, and intelligence/imagination—that are scored from 0 to 5, where 1=imprecise, 2=slightly imprecise, 3=ordinary, 4=slightly precise, and 5=very precise. The Cronbach α reliability coefficient judging the internal consistency of each scale ranged between .67 and .83, and the factor loading ranged between .61 and .83, indicating convergent validity. The correlation between the IPIP-15 and the personality scales corresponding to the original IPIP-50 ranged between .81 and .88, which indicated that the convergent validity was acceptable [42].

Participants’ experience in the use of technology, networking, and eHealth literacy were also assessed. The question about experience with the use of technology and networking was answered by subjects based on their past experience (ie, whether they had experience using the internet and downloading and using mobile apps). The question about eHealth literacy was assessed using the eHealth Literacy Scale (eHEALS), which is an 8-item measure that assesses the participant’s internet use and search skills, ability to evaluate online content, and confidence in their internet-searching abilities. This scale is scored with 4 points, with the options being entirely disagree, disagree, agree, and strongly agree, which are scored 1, 2, 3, and 4, respectively. The internal consistency Cronbach α reliability coefficient for each item is .88, and the factor load ranges between .60 and .84 [43,44].

Experience with and acceptance of robots and pets included the acceptability of choosing a robot or pet as a companion object in later life, the type of companionship robot desired/favored, and past experience with keeping pets. Among these indices, the question about the acceptability of robots/pets was answered based on the response, “Acceptability of choosing a robot or pet as a companion object in later life.” It was scored from 0 to 10, with 0 representing completely unacceptable and 10 representing quite acceptable, and the higher the score, the higher the degree of acceptability. In addition, when the type of companion robots desired/favored was also evaluated, subjects were requested to choose the types and functions of the robots according to their preferences or needs. The choices included the companionship robot’s services (eg, assisting with family tasks, health monitoring, safety monitoring), skills (eg, juggling, dancing, singing), interaction (eg, chatting, storytelling, news reporting, joke telling, providing child-like dialogue), expression, and appearance (eg, resembling an animal, human infant or adult, or nonbiological form), as well as other functions.

### Procedure

**Figure 1:**
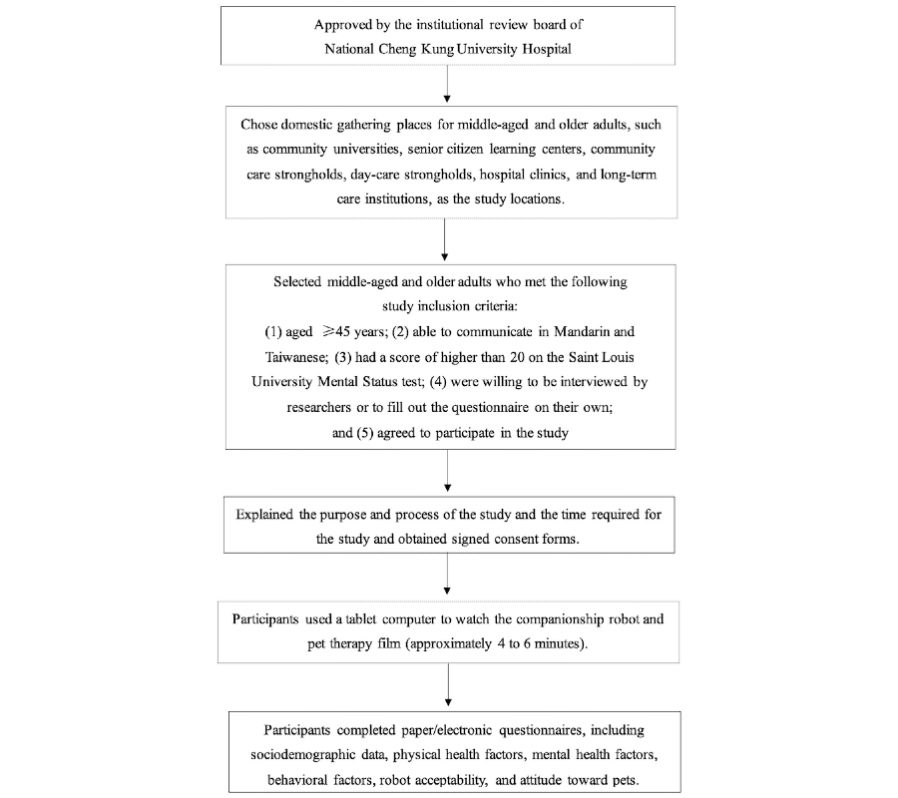
Research and data collection flowchart.

The study was conducted between May and June 2018. A cross-sectional survey research method was used to survey the degree of acceptance and factors related to the choices made by middle-aged and older adults in Taiwan of a robot or pet as their companion object in later life. This study was approved by the institutional review board (IRB) of National Cheng Kung University Hospital in Taiwan (No. A-ER-105-509). The study collection methods and procedures are shown in Figure 1.

With the consent of the IRB of National Cheng Kung University Hospital in Taiwan, middle-aged and older adults in Taiwan were invited to participate in the study, and gathering places for the middle-aged and older adults, such as community universities, senior citizen learning centers, community care strongholds, and day-care strongholds, were chosen as places to distribute questionnaires. Middle-aged and older adults who met the following inclusion criteria were selected: (1) aged ≥45 years, (2) able to communicate in Mandarin and Taiwanese, (3) had a Saint Louis University Mental Status test score higher than 20, (4) willing to be interviewed by the researchers or to fill out the questionnaire on their own, and (5) agreed to participate in the study and sign a consent form. The researchers explained to the subjects the study’s purpose, process, and duration. After signing the consent form, the subjects used a tablet computer to watch a companionship robot film, after which they completed a questionnaire.

A companionship robot film was used in the study to provide a brief introduction to each type of companionship robot and was explained by the researchers at the time it was played. The film included robots with various physical characteristics (Multimedia Appendix 1), such as resembling an adult, an infant, an animal, or a nonbiological object, and presented the different functions of each robot, such as assisting with family tasks, health monitoring, safety monitoring, and other services, and the content of the robots’ interactions with users, such as chatting, reporting news, reporting weather, singing, dancing, and making various expressions, among other functions.

### Statistics

The descriptive statistics included an analysis of the sociodemographic variables, physical health factors, past experience with keeping pets, and experience in the use of technology as they related to variables such as the acceptability of choosing a robot or pet as a companion object in later life using a *t* test and a 1-way analysis of variance. Differences among the sociodemographic variables, physical health factors, past experience with keeping pets, and experience with the use of technology, as well as other variables related to the elderly, were verified using the chi-square test among 4 groups of robot/pet preferences. The analysis of the correlation between the continuous variables was related to the Pearson correlation and included age, number of years living alone, IPIP-15 score, CES-D score, ISSB score, eHEALS score, and the level of acceptance of a robot or pet as a companion object in later life. A multiple regression analysis was used to analyze the between-variable correlations, such as gender, age, education level, living alone or not, retirement status, number of comorbidities, ISSB score, eHEALS score, IPIP-15 score, and acceptance of either robots or pets. Finally, a multinomial logistic regression analysis was used to analyze the predictive power of different groups of robot or pet acceptability in the middle-aged and older adults based on the following variables: (1) both robots and pets were highly acceptable (HH), (2) preferred choice was a robot (HL), (3) preferred choice was a pet (LH), and (4) neither robots nor pets were acceptable (LL).

## Results

### Descriptive Analysis of the Subjects’ Basic Data

For the purpose of discussing the degree to which the middle-aged and older adults preferred a robot or pet as their companion object in later life in Taiwan, the questionnaires were distributed at 6 community care strongholds, 5 community centers, and 3 large-scale activities related to respecting the elderly and Mother’s Day events in the north, middle, and southern parts of Taiwan. A total of 273 subjects who met the inclusion criteria were selected out of 300 middle-aged and older adults living in the community who were aged ≥45 years, and a total of 240 valid questionnaires were obtained after those with missing data or incorrect answers (n=33) were removed. The minimum and maximum ages of the participants were 45 years and 94 years, respectively. The average age was 60.68 years, and there were 172 (71.7%) female participants and 68 (28.3%) male participants. The majority of participants were highly educated (183/240, 76.2%), had a partner (170/240, 70.8%), lived in the city (214/240, 89.2%), did not live alone (215/240, 89.6%), lived with children (221/240, 92.1%), had no experience with using robots (197/240, 82.1%), had experience using the internet (196/240, 81.7%), could download and use an app (184/240, 76.7%), had experience with keeping pets (152/240, 63.6%), and had no experience with animal-assisted therapy (226/240, 94.2%). The subjects reported an average of 0.65 chronic conditions and an average self-reported health score of 3.53 (out of 5). On average, subjects’ level of acceptance of robots and pets was 5.69 points and 4.72 points, respectively. A detailed chart of the data distribution is shown in Table 1.

**Table 1:** Basic sociodemographic data of participants.

Characteristic			Preference of robot or pet as a companion				
		Full sample (N=240)	Both are highly acceptable^a^ (n=81, 33.8%)	Prefer robot (n=42, 17.5%)	Prefer pet (n=56, 23.3%)	Neither is acceptable (n=61, 25.4%)	Verification value (*F*/χ^2^)
**Age (years), mean (SD)**		60.68 (10.496)					13.65
	45-54	85 (35.4)	37 (45.7)	8 (19.0)	22 (39.3)	18 (29.5)	
	55-64	73 (30.4)	21 (25.9)	13 (31.0)	20 (35.7)	19 (31.1)	
	65-74	53 (22.1)	14 (17.7)	14 (33.3)	10 (17.9)	15 (24.6)	
	≥75	29 (12.1)	9 (11.1)	7 (16.7)	4 (7.1)	9 (14.8)	
**Gender, n (%)**							1.34
	Male	68 (28.3)	22 (27.2)	12 (28.6)	19 (33.9)	15 (24.6)	
	Female	172 (71.7)	59 (72.8)	30 (71.4)	37 (66.1)	46 (75.4)	
**Education level, n (%)**							9.41
	Below primary school	57 (23.8)	15 (18.5)	15 (35.7)	11 (19.6)	16 (26.2)	
	Secondary school/senior high school (higher vocational school)	73 (30.4)	23 (28.4)	8 (19.0)	19 (33.9)	23 (37.7)	
	University and above	110 (45.8)	43 (53.1)	19 (45.2)	26 (46.4)	22 (36.1)	
**Marital status, n (%)**							4.48
	Unmarried/widowed/no partner	70 (29.2)	18 (22.2)	16 (38.1)	15 (26.8)	21 (34.4)	
	Married or has a partner	170 (70.8)	63 (77.8)	26 (61.9)	41 (73.2)	40 (65.6)	
**Residence, n (%)**							0.866^b^
	City	214 (89.2)	71 (33.2)	38 (90.5)	49 (87.5)	56 (91.8)	
	Village	26 (10.8)	10 (12.4)	4 (9.5)	7 (12.5)	5 (8.2)	
**Type of dwelling, n (%)**							10.19
	House	109 (45.4)	42 (51.9)	20 (47.6)	23 (41.1)	24 (39.3)	
	Apartment building without elevator	39 (16.3)	13 (16.0)	4 (9.5)	6 (10.7)	16 (26.2)	
	Apartment building with elevator	92 (38.3)	26 (32.1)	18 (42.9)	27 (48.2)	21 (34.4)	
**Lives alone, n (%)**							4.244^b^
	Yes	25 (10.4)	7 (8.6)	5 (11.9)	3 (5.4)	10 (16.4)	
	No	215 (89.6)	74 (91.4)	37 (88.1)	53 (94.6)	51 (83.6)	
Number of children, mean (SD)		2.16 (1.117)					
Number of years living alone (years), mean (SD)		0.90 (3.491)	0.69 (3.204)	1.55 (4.910)	0.73 (3.419)	0.87 (2.699)	0.62 (1.146)^b^
**Lives with children, n (%)**							1.146^b^
	Yes	221 (92.1)	75 (92.6)	37 (88.1)	52 (92.9)	57 (93.4)	
	No	19 (7.9)	6 (7.4)	5 (11.9)	4 (7.1)	4 (6.6)	
**Type of occupation, n (%)**							10.05
	Unskilled	76 (31.7)	20 (24.7)	16 (38.1)	14 (25.0)	26 (42.6)	
	Semiskilled or skilled	87 (36.3)	32 (39.5)	12 (28.6)	20 (35.7)	23 (37.7)	
	Professional/senior managers	77 (32.1)	29 (35.8)	14 (33.3)	22 (39.3)	12 (19.7)	
**Retirement status, n (%)**							9.40*
	Retired	118 (49.2)	32 (39.5)	27 (64.3)	24 (42.9)	35 (57.4)	
	Employed	122 (50.8)	49 (60.5)	15 (35.7)	32 (57.1)	26 (42.6)	
Number of chronic diseases, mean (SD)		0.65 (0.878)	0.43 (0.670)	1.02 (1.047)	0.70 (0.807)	0.66 (0.981)	4.434**
Self-rated health (1 to 5 points), mean (SD)		3.53 (0.731)	3.58 (0.756)	3.50 (0.672)	3.43 (0.828)	3.59 (0.642)	0.644
Self-rated financial status (1 to 5 points), mean (SD)		3.83 (0.062)	3.88 (0.509)	3.81 (0.594)	3.75 (0.745)	3.85 (0.654)	0.501
**Has experience using robots, n (%)**							3.16
	Yes	43 (17.9)	18 (22.2)	7 (16.7)	6 (10.7)	12 (19.7)	
	No	197 (82.1)	63 (77.8)	35 (83.3)	50 (89.3)	49 (80.3)	
**Has experience using the internet, n (%)**							7.80
	Yes	196 (81.7)	65 (80.2)	30 (71.4)	52 (92.9)	49 (80.3)	
	No	44 (18.3)	16 (19.8)	12 (28.6)	4 (7.1)	12 (19.7)	
**Experience using apps, n (%)**							2.51
	No experience with using apps/not able to download	56 (23.3)	20 (35.7)	13 (23.2)	12 (21.4)	11 (19.6)	
	Can download and use apps	184 (76.7)	61 (75.3)	29 (69.0)	44 (78.6)	50 (82.0)	
**Has experience keeping pets, n (%)**							20.55***
	Yes	152 (63.3)	63 (77.8)	18 (42.9)	40 (71.4)	31 (50.8)	
	No	88 (36.7)	18 (22.2)	24 (57.1)	16 (28.6)	30 (49.2)	
**Animal-assisted therapy experience, n (%)**							1.806^b^
	Has no animal care experience	226 (94.2)	76 (93.8)	40 (95.2)	51 (91.1)	59 (96.7)	
	Has animal care experience	14 (5.8)	5 (6.2)	2 (4.8)	5 (8.9)	2 (3.3)	
**IPIP-15^c^ >score, mean (SD)**							
	Extroversion	11.51 (2.626)	11.69 (2.391)	11.55 (2.487)	11.50 (2.683)	11.25 (2.987)	0.335
	Friendliness	11.78 (2.141)	11.77 (2.260)	11.76 (2.034)	12.02 (2.244)	11.59 (1.978)	0.390
	Rigorousness	11.90 (2.405)	11.57 (2.617)	12.19 (2.287)	12.29 (2.078)	11.80 (2.455)	1.235
	Emotional stability	11.28 (2.746)	10.91 (2.651)	11.71 (3.263)	11.34 (2.345)	11.41 (2.831)	0.884
	Intelligence/imagination	9.72 (2.604)	9.81 (2.393)	9.43 (2.881)	10.09 (2.678)	9.44 (2.617)	0.815
CES-D^d^ score, mean (SD)		5.93 (5.330)	6.07 (5.422)	6.10 (5.938)	5.79 (5.098)	5.74 (5.092)	0.072
Social participation, mean (SD)		2.25 (2.124)	2.14 (2.223)	2.81 (2.211)	2.05 (2.211)	2.18 (1.812)	1.234
Leisure activities, mean (SD)		7.85 (2.147)	8.01 (2.009)	7.93 (2.005)	7.75 (2.250)	7.69 (2.349)	0.325
Social support, mean (SD)		24.88 (4.462)	24.67 (4.693)	24.88 (4.743)	25.05 (4.020)	25.00 (4.431)	0.104
eHealth Literacy Scale score, mean (SD)		21.65 (7.773)	22.04 (7.825)	21.36 (8.316)	23.43 (6.494)	19.69 (8.123)	2.400
**Acceptability^a^ (0 to 10 points), mean (SD)**							
	Robot	5.69 (3.142)					
	Pet	4.72 (3.564)					

^a^Acceptability was deemed to be high or low if it was higher or lower, respectively, than the average points of acceptability.

^b^Because the number of people who had few expectations was less than 5, the test was conducted using Fisher exact test, and the results showed no significant differences.

^c^IPIP-15: 15-item International Personality Item Pool big 5 personality scale.

^d^CES-D: Center for Epidemiologic Studies Depression Scale.

*P<.05; **P<.01; and ***P<.001.

### Factors Associated With Intention to Use a Robot or Pet as Their Companion Object in Later Life

The variables related to acceptance of robots and pets were divided into 4 groups: both robots and pets were highly acceptable (HH), preferred a robot (HL), preferred a pet (LH), and neither robots nor pets were acceptable (LL). The level of acceptability was deemed to be high if it was higher than average and was deemed to be low if it was lower than the average. As Table 1 shows, 33.8% (81/240) of the subjects reported both robots and pets to be acceptable (HH), 17.5% (42/240) preferred a robot (HL), 23.3% (56/240) preferred a pet (LH), and 25.4% (61/240) reported neither robots nor pets to be acceptable (LL). The results of the study showed that retirement status (χ^2^_3_=9.40, *P=*.024), experience with keeping pets (χ^2^_3_=20.55, *P=*.000), and the number of comorbidities (*F*_3,236_=4.43, *P=*.005) were all significantly associated with the 4 acceptance groups. However, none of the psychological measures, including personality traits, CES-D score, and social participation, were associated with the preference for robots or pets as their companion.

Results of the multinomial logistic regression analysis used to analyze the variables in terms of their predictive power on the 4 groups of robot/pet acceptability among the middle-aged and older adults is presented in Table 2. The results showed that those with more comorbidities were 1.688 times more likely to fall into the HL group than into the LL group (*P=*.048); those who could download and use an app were 0.170 times more likely to fall into the HL group than the LL group as compared with those who had not used or downloaded apps (*P=*.022). Those who could download and use apps were 0.159 times more likely to fall into the HL group than into the LL group as compared with those who had not used or downloaded apps (*P=*.012). Those with experience with keeping pets were 3.527 times more likely to fall into the HH group than into the LL group as compared with those who had no experience with keeping pets (*P=*.002). Those who had experience with keeping pets were 2.498 times more likely to fall into the LH group than into the LL group as compared with those with no experience in keeping pets (*P=*.034). Those with a higher score on eHEALS were 1.084 times more likely to fall into the HH group than into the LL group (*P=*.039). Those with higher scores on eHEALS were 1.139 times more likely to fall into the HL group than into the LL group (*P=*.005). Finally, those with higher scores on eHEALS were 1.100 times more likely to fall into the LH group than into the LL group (*P=*.020).

**Table 2:** Multinomial logistic regression analysis of the degree of acceptance of robots and pets by middle-aged and older adults (N=240).^a^

Characteristic		Preference of robot or pet as a companion		
		Both robots and pets are highly acceptable (n=81, 33.8% [OR])	Prefer robots (n=42, 17.5% [OR])	Prefer pets (n=56, 23.3% [OR])
**Age, years** **(reference: ≥75 years old)**				
	45-54	1.392	0.982	1.849
	55-64	0.896	1.953	1.649
	65-74	1.066	2.234	1.129
Number of comorbidities		0.877	1.688*	1.348
Lives alone		0.484	0.664	0.275
Male gender		0.863	1.173	1.646
**Education level (reference: above university)**				
	Below primary school	0.939	0.645	1.593
	Secondary school/senior high school (higher vocational school)	0.759	0.370	1.080
Retired		0.555	1.015	0.771
Has experience using robots		0.933	0.734	0.358
Has experience using the internet		0.471	0.368	3.350
Can download and use apps		0.271	0.170*	0.159*
Has experience with keeping pets		3.527**	0.784	2.498*
Social support score		0.969	0.953	0.977
eHealth Literacy Scale score		1.084*	1.139**	1.100*

^a^Contrast group: neither robots nor pets are acceptable (n=61; 25.4%).

*P<.05; **P<.01; and ***P<.001.

### Subjects’ Preferences for Companionship Robot Functions

This section discusses the participants’ preferences for the functions of the companionship robot as their companion object in later life. The subjects filled out questionnaires regarding their preferences or needs for functions of the companionship robot. The functions of the companionship robot included (1) family services, (2) health status monitoring, (3) safety monitoring, (4) skill and recreation-type functions (eg, juggling, dancing, singing, storytelling, news reporting, joke telling, the ability to make various expressions), and (5) interactive functions (eg, chatting, providing child-like dialogue).

The results of the study showed that the functions of the companionship robot that the subjects most desired were the skill and recreation-type functions (211/240, 87.9%), followed by family services (185/240, 77.1%), interactive functions (160/240, 66.7%), health status monitoring (147/240, 61.3%), and safety monitoring (144/240, 60.0%), as shown in Table 3. By analyzing the correlation between the sociodemographic characteristics and the function selection for the companionship robot, it was found that in addition to skill and recreation-type functions (60/68, 88.2%) and family services (50/68, 73.5%), male subjects desired health status monitoring functions (46/68, 67.6%) of the companionship robot more than its interactive functions (44/68, 64.7%). Female subjects desired the safety monitoring functions (103/172, 59.9%) of the companionship robot more than its health status monitoring functions (101/172, 58.7%). Among these, although the differences were not statistically significant, the middle-aged and older female subjects still preferred family service–type robots and interactive function–type robots more than the male subjects.

**Table 3:** Analysis of the preference of middle-aged and older adults for the companionship robot functions (N=240).

Characteristic			Robot functions								
			Skill and recreation-type functions		Family services		Interactive functions		Health status monitoring		Safety monitoring
Full sample, n (%)			211 (87.9)		185 (77.1)		160 (66.7)		147 (61.3)		144 (60.0)
**Gender**											
	Male (n=68), n (%)	60 (88.2)		50 (73.5)		44 (64.7)		46 (67.6)		41 (60.3)	
	Female (n=172), n (%)	151 (87.8)		135 (78.5)		116 (67.4)		101 (58.7)		103 (59.9)	
	χ^2^	0.009		0.678		0.164		1.636		0.003	
**Age**											
	45-54 years (n=85), n (%)	73 (85.9)		74 (87.1)		59 (69.4)		61 (71.8)		60 (70.6)	
	55-64 years (n=73), n (%)	66 (90.4)		60 (82.2)		46 (63.0)		46 (63.0)		43 (58.9)	
	≥65 years (n=82)	72 (87.8)		51 (62.2)		55 (67.1)		40 (48.8)		41 (50.0)	
	χ^2^	0.760		16.156***		0.733		9.427**		7.424*	
**Residential status**											
	Living alone (n=25), n (%)	23 (92.0)		15 (60.0)		16 (64.0)		15 (60.0)		11 (44.0)	
	Not living alone (n=215), n (%)	188 (87.4)		170 (79.1)		144 (67.0)		132 (61.4)		133 (61.9)	
	χ^2^	0.438		4.610*		0.089		0.018		2.977	
**Marital status**											
	Have a partner (n=170), n (%)	153 (90.0)		134 (78.8)		113 (66.5)		111 (65.3)		107 (62.9)	
	Have no partner (n=70), n (%)	58 (82.9)		51 (72.9)		47 (67.1)		36 (51.4)		37 (52.9)	
	χ^2^	2.381		0.999		0.010		4.016*		2.101	

*P<.05; **P<.01; and ***P<.001.

According to the results of the analysis on residential status and preferences for functions of the companionship robot, 64% (16/25) of the people living alone had a greater preference and demand for interactive functions, which took second place among those living alone in terms of the desired functions of the companionship robot. Among these subjects, whether or not they lived alone was significantly related to the preference for family service (ie, housework) functions (*P=*.032). The results for choosing skill and recreation-type functions for the companionship robot by those living alone were not statistically significant, but there was still a tendency toward choosing this type of robot.

The results of the analysis on the marital status and the preference for the companionship robot functions showed that for subjects who had no partner, the level of preference and demand for safety monitoring functions was higher than for health status monitoring functions, and having a partner was significantly related to the choice of health status monitoring functions (*P=*.045).

The subjects were divided into 3 age groups: 45 to 54 years, 55 to 64 years, and ≥65 years. By analyzing the choices made by the elderly in each age group for the functions of the companionship robot, it was found that the subjects ranging in age from 45 to 54 years mainly preferred the family service–type robot followed by the skill and recreation-type robot. However, subjects who were aged 55 to 64 years and over 65 years all chose the skill and recreation-type robot, including the functions of juggling, dancing, singing, storytelling, news reporting, joke telling, and the ability to make various expressions, followed by the family service–type robot. The different ages were significantly related to the choice of family service (*P*<.001), health status monitoring (*P=*.009), and safety monitoring (*P=*.026) functions of the companionship robot.

### Subjects’ Preferences for the Appearance of the Companionship Robot

This section discusses the preferences of the middle-aged and older adults for the appearance of the companionship robot as their companion object in later life. The subjects filled out a questionnaire according to their preferences or requirements for the appearance of the companionship robot, which included animal, infant, adult, and nonbiological or other form.

It was found that the appearance of the companionship robot that the subjects most desired/preferred was one resembling an animal (94/240, 39.2%), followed by one resembling an adult (72/240, 30.0%), an infant (43/240, 17.9%), and a nonbiological or other form (21/240, 8.9%). The results are shown in Table 4. The analysis of the correlation between the sociodemographic characteristics and the choice of the appearance of the companionship robot showed that male subjects preferred a companionship robot that resembled an adult (29/68, 42.6%), followed by an animal-like appearance (23/68, 33.8%), where gender was found to be significantly related to the preference for the companionship robot to look like a human adult (*P=*.007). The correlations between residential status and marital status with choice of robot appearance did not reach statistical significance, but living alone and with a partner had the same ranking as the full sample in terms of this preference. Regardless of age, the appearance of animals was the most popular choice of robot appearance. The second most preferred robot appearance was that of a human adult among subjects who were aged 45 to 54 years old and those aged 55 to 64 years; subjects who were ≥65 years preferred the infant- and adult-like appearances equally.

**Table 4:** Analysis of the preference of the middle-aged and older adults for the appearance of the companionship robot (N=240).

Characteristic		Preferred appearance of companionship robot			
		Animal	Adult	Infant	Other
Full sample, n (%)		94 (39.2)	72 (30.0)	43 (17.9)	21 (8.9)
**Gender**					
	Male (n=68), n (%)	23 (33.8)	29 (42.6)	7 (10.3)	4 (5.9)
	Female (n=172), n (%)	71 (41.3)	43 (25.0)	36 (20.9)	17 (9.9)
	χ^2^	1.137	7.227**	3.749	0.977
**Age**					
	45-54 years (n=85), n (%)	36 (42.4)	13 (15.3)	28 (32.9)	8 (9.4)
	55-64 years (n=73), n (%)	29 (39.7)	12 (16.4)	26 (35.6)	7 (9.6)
	≥65 years (n=82), n (%)	29 (35.4)	18 (22.0)	18 (22.0)	6 (7.3)
	χ^2^	0.869	3.976	1.414	0.322
**Residential status**					
	Living alone (n=25)	73 (42.9)	51 (30.0)	27 (15.9)	16 (9.4)
	Not living alone (n=215)	21 (30.0)	21 (30.0)	16 (22.9)	5 (7.1)
	χ^2^	3.485	0.000	1.640	0.320
**Marital status**					
	Have a partner (n=170)	12 (48.0)	8 (32.0)	4 (16.0)	1 (4.0)
	Have no partner (n=70)	82 (38.1)	64 (29.8)	39 (18.1)	20 (9.3)
	χ^2^	0.914	0.053	0.070	0.789

**P<.01

## Discussion

### Principal Findings

The main purpose of this study was to discuss the level of acceptance of middle-aged and older adults toward a robot or pet as their companion object in later life; to understand the correlation between sociodemographic variables, physical health, mental health, behavioral factors, and preferences for either a robot or pet as a companion object in later life; and to further analyze the needs and preferences of the subjects for the functions and appearance of a companionship robot. The results of the community survey showed that the level of acceptance of subjects in the community toward a pet as their companion object in later life was significantly correlated with their age, with a higher age being associated with a lower average score for acceptance of a pet. Acceptance of pets was significantly correlated with education level, type of occupation, retirement status, number of comorbidities, past experience with keeping pets, an extroverted personality, an intellectual/imaginary personality, and eHealth literacy. In terms of the acceptance toward robots, there were no significant differences in the level of acceptance based on age group. The level of acceptance toward robots was only significantly related to the type of dwelling the subject lived in. No correlation between the acceptance toward robots and other sociodemographic variables, or physical health, mental health, or behavioral factors was observed in this study.

According to the acceptability score, the level of acceptance of the middle-aged and older adults for choosing between a robot or pet as their companion object in later life was divided into 4 groups: both are highly accepted (HH), preferred a robot (HL), preferred a pet (LH), and neither was acceptable (LL). When the average acceptability score was used as the grouping standard, there were significant differences in terms of retirement status, number of comorbidities, and past experience with keeping pets among each group of subjects. The results of the multinomial logistic regression analysis on the key variables showed that the number of comorbidities, experience with keeping pets, experience with using apps, and eHealth literacy had significant predictive power for the level of acceptability among all of the groups; however, gender, intergenerational differences, education level, whether or not subjects lived alone, social support, and past experience with keeping pets had no significant impact on the level of acceptability among the various groups.

According to the theory of planned behavior, attitude, subjective norms, and perceived behavioral control are the 3 factors that determine behavior [28]. In the research, although the reasons for the significant variables were not further explored, we can infer that personality, education level, type of occupation, and past experience with keeping pets, which can affect the experience of life, might be related to personal attitude. The type of dwelling in which a person resides may affect their preference for robots or pets. It may be related to subjective norms; after all, sometimes living conditions such as neighbors or house size might restrict one from keeping pets. On the other hand, age (related to one’s functional ability to keep pets), retirement status (ie, how much leisure time someone has), number of comorbidities, experience with using apps, and eHealth literacy might be related to perceived behavioral control. Overall, the results showed that preferences for robots and pets among middle-aged and older adults conformed to the theory of planned behavior.

By analyzing the needs and preferences of middle-aged and older adults for the companionship robot, it was found that these individuals desired/favored robot skills such as juggling, dancing, singing, storytelling, news reporting, joke telling, or the ability to make various expressions, followed by family service, interactive, health status monitoring, and safety monitoring functions. In terms of the appearance of the companionship robot, the middle-aged and older adults preferred the robot to look like an animal, followed by it having an adult-like appearance, infant-like appearance, and nonbiological or other appearance, in that order. By further analyzing the impact of sociodemographic characteristics on the preferences for the companionship robot, it was found that whether the subjects lived alone or not significantly affected the choice of the family service function of the robot, and whether there was a partner or not also significantly affected whether the subjects chose the health status monitoring function of the robot. Male subjects showed a greater preference for an adult-like appearance in the robot than did female subjects, and the difference was statistically significant. Female subjects preferred an animal-like appearance to the robot over an adult-like appearance.

### Comparison With Prior Work

Most studies on the appearance of robots have pointed out that elderly individuals prefer less human-looking robots such as pet-like robots, which have been widely used in the past to care for the elderly and are highly accepted by them [12,13,45]. Studies in Japan showed that the robotic seal pet PARO, because its seal-like appearance was unfamiliar to people, did not lead to an unreal sense of interaction with a fake animal and was easily accepted by the elderly [46]. A study discussing robots in the daily lives of the elderly in Taiwan pointed out that older adults were more likely to accept robot pets of traditional pet animals, such as cats or dogs, because the elderly associated the robots with animals they were familiar with, and those who had no experience with keeping pets wanted pet-like robots as pets [47]. A study discussing the needs of the elderly for the companionship robot when they entered the “empty-nest” period indicated that the appearance of a future companionship robot needed to be based on human life experiences. For example, the Hug is a robot that allows the elderly to maintain social and affective interactions by communicating closely with their families. It was designed to look like a human offering a hug and has specific types of communication functions [48]. The SenseChair is a robot that was designed to look like a “chair” that the elderly are familiar with in their daily life [49]. In terms of the robot’s functions, the main purpose of using a home-based robot was to obtain the “home service” function, following by providing assistance to people with mobility disabilities, home security management, remote monitoring, emotional pacification, and so on. The study also pointed out that companionship robots with more social functions are more likely to elicit expressions and responses from the elderly and to promote the participation of the elderly in social interactions and enhance the quality of their interactions [19,50].

### Limitations and Future Work

There were some limitations to this study. First, the research tool was self-reported, and the subjects’ understanding of the questions in the questionnaire and their personal perception of their own situation all affected their answers. The subjects’ answers might have been affected by extrinsic factors beyond their control such as mood and social expectations, so the results of the inventory might have been different from their actual situation, resulting in measurement errors in the results of the study. Second, the sampling sites for the study were gathering places for the elderly, such as community care strongholds and community centers, and at activities intended to respect the elderly and as part of Mother’s Day events. The subjects were middle-aged and older adults who were more active and had the intention and ability to go out, so the results of the study are limited in terms of extrapolation. Third, the sample size and representativeness of the subjects were not as good as probability sampling, which affected any inferences that could be made based on the results, although the study areas covered the northern, central, and southern parts of Taiwan to increase the robustness of the results of the study. Fourth, this research is the first attempt at understanding companionship preferences toward pets and robots among the elderly in the community in Taiwan. Therefore, cultural factors were not considered. Similarly, because there have been few studies of this topic abroad [29], it is difficult to know whether there are similarities or differences between cultures, which is an expectant direction for future work.

In spite of these limitations, this study is a rare survey of the perceptions of elderly individuals living in the community toward companionship robots. The subjects in this study were aged 45 to 94 years and included active middle-aged and older adults living in the community in the northern, central, and southern parts of Taiwan. It is the only study in Taiwan to compare pets and robots from the point of view of choosing them as a companion object. From the results of the study, we can preliminarily understand the current situation and preferences of the needs for companionship in middle-aged and older adults living in the community. In addition to contributing to the literature, middle-aged and older adults at home can find a suitable companion object in later life based on their sociodemographic characteristics. First-line community practitioners can also design care projects for middle-aged and older adults living with different needs and backgrounds and provide a future implementation plan for the welfare system, as well as provide empirical evidence for policy promotion and the development of science- and technology-related industrial products.

### Conclusion

The key findings of this study are as follows. First, variables such as age, education level, type of dwelling, occupation, retirement status, number of comorbidities, experience with keeping pets, experience with using apps, and eHealth literacy significantly affected the degree of acceptance of a robot or pet as a companion object in later life. Community practitioners working with middle-aged and older adults could plan curricula according to the different backgrounds and characteristics of the population of interest to develop care projects for middle-aged and older adults based on their different needs and backgrounds and help them to select appropriate companion objects in later life. Second, the study found that eHealth literacy significantly affected the degree of acceptance of robots and pets in the middle-aged and older adults as well as the type of functions desired in a companion robot. Those with higher eHealth literacy scores were more likely to respond that both robots and pets were acceptable as companion objects. This indicates that those with better eHealth literacy are more likely to choose a robot or pet as their companion object in later life and that eHealth literacy is significantly associated with age. First-line staff or policy makers in relevant fields can conduct eHealth literacy promotion courses for middle-aged and older adults in order to facilitate the implementation of relevant plans. Third, in terms of the development of companionship robot products, middle-aged and older women generally preferred animal-like robots as companion objects in later life, while men preferred an adult human-like robot. In terms of functions, middle-aged and older adults in the community are more likely to need a companionship robot that has functions including dancing, singing, storytelling, or news reporting. Whether or not they live alone or with a partner also affects their preferred robot functions, so technology-related industries should consider designing products to suit the needs of different target groups. It would be useful to increase the number of study samples or select specific groups to carry out intensive studies. Heterogeneous populations in residential institutions or extension of the sampling sites could make the results more generalizable. In addition, qualitative study methods such as in-depth interviews would help lead to an understanding of the needs of middle-aged and older adults in terms of a companion in later life. Study methods attaching equal importance to quality and quantity in the future could potentially better reflect the current needs of middle-aged and older adults.

## Appendix

Multimedia Appendix 1 Different types of robots.

Multimedia Appendix 2 Questionnaires.

## Abbreviations

CES-D: Center for Epidemiologic Studies Depression Scale
eHEALS: eHealth Literacy Scale
ICT: information and communications technology
IPIP: International Personality Item Pool
IPIP-15: 15-item version of the International Personality Item Pool
IRB: institutional review board
ISSB: Inventory of Socially Supportive Behavior
mHealth: mobile health
