# Supplementary material for: Needs and Preferences of Middle-Aged and Older Adults in Taiwan for Companion Robots and Pets: Survey Study
Source: J Med Internet Res. 2021 Jun 11;23(6):e23471. doi: 10.2196/23471 (PMC8386361; doi:10.2196/23471)
Supplement: Multimedia Appendix 1 [file jmir_v23i6e23471_app1.docx]

Appendix. Different types of robots.

| Adult appearance | 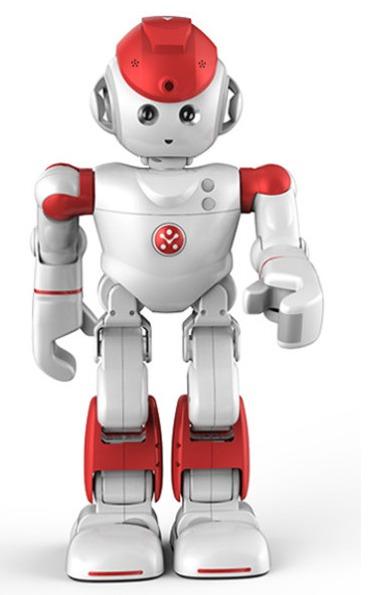  *ALPHA* | 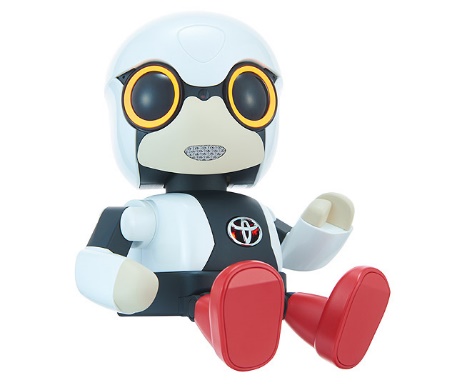  *KIROBO mini* | | 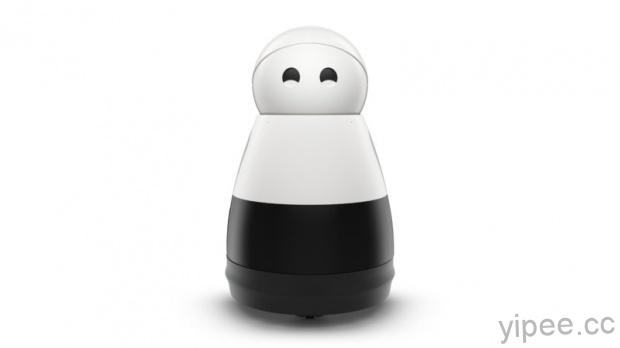  *Kuri* |
| --- | --- | --- | --- | --- |
| Infant appearance | 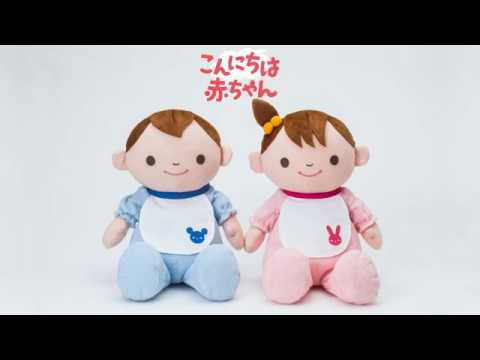  *電子孫子* | 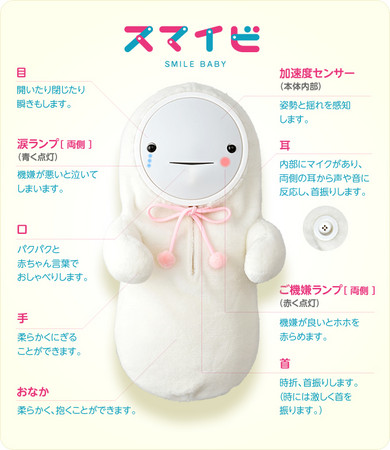  *Smile Baby* | | |
| Animal appearance | 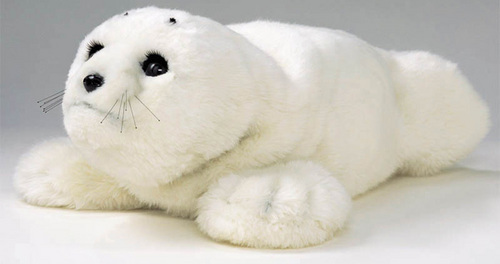  *Paro* | 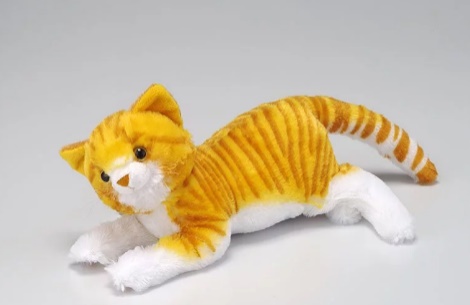*DX2* | | 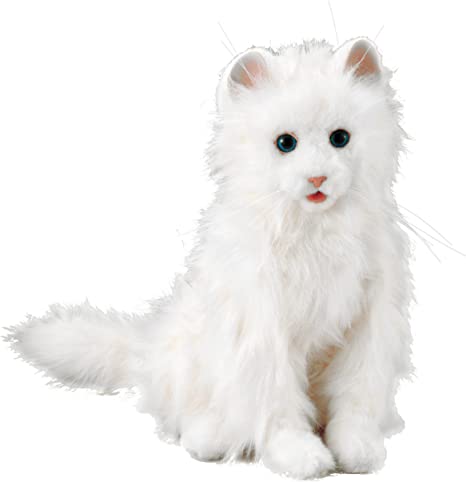*ねこセレブ* |
|  | 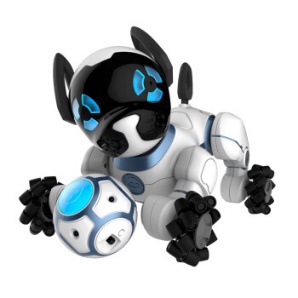  *CHiP* | 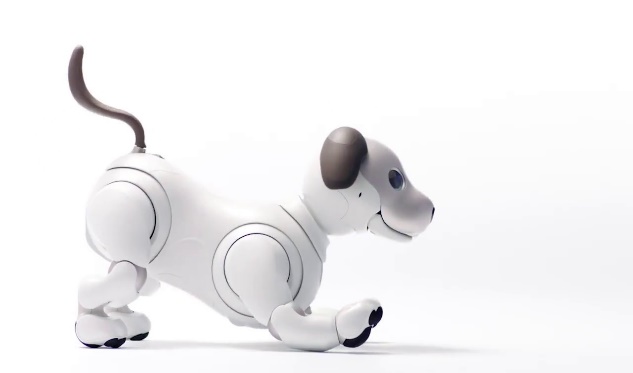  *Aibo* | |  |
| Non-biological appearance | 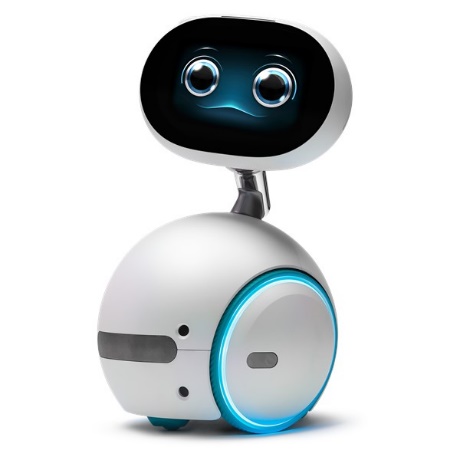*Zenbo* | 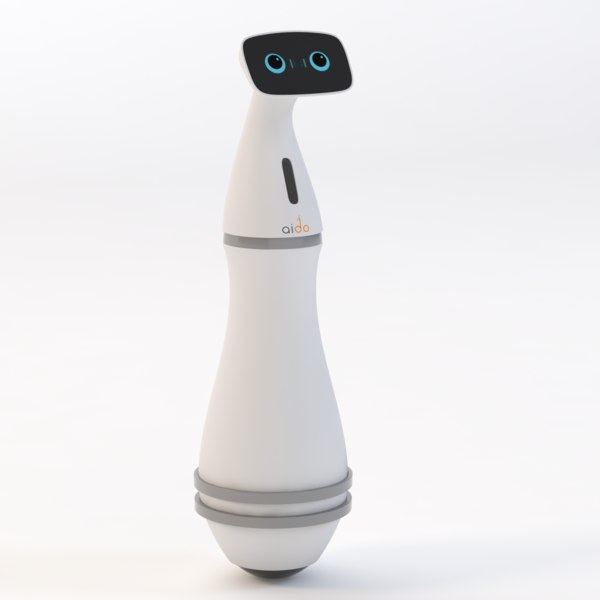  *Aido* | | *Robelf* 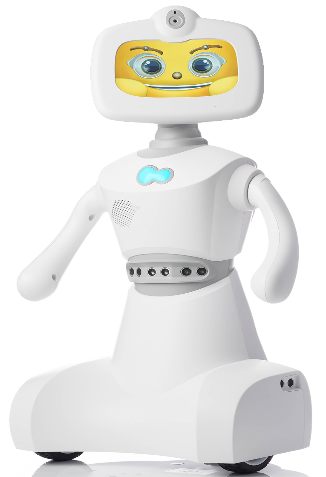 |
|  | 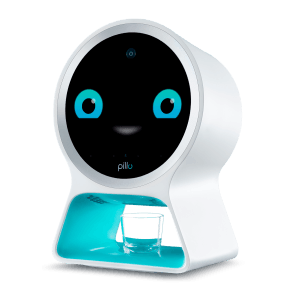  *Pillo* | 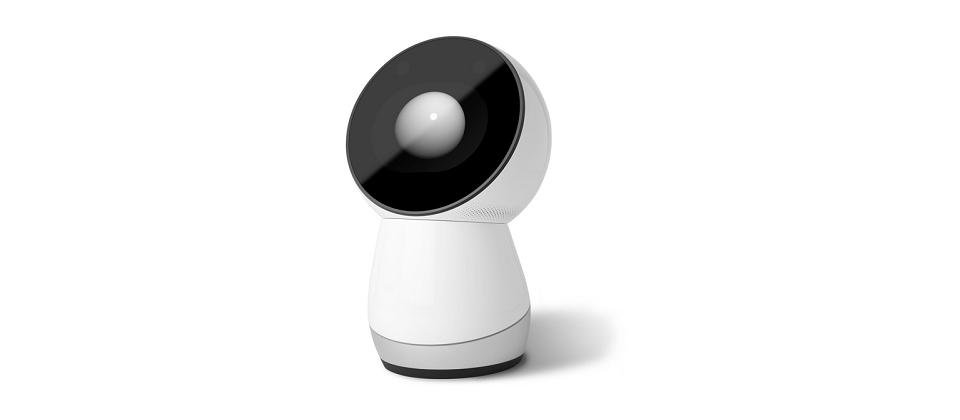  *Jibo* | 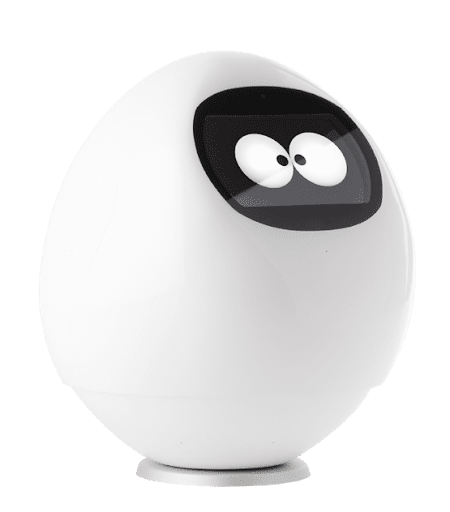  *Tapia* | |
