# Supplementary material for: Needs and Preferences of Middle-Aged and Older Adults in Taiwan for Companion Robots and Pets: Survey Study
Source: J Med Internet Res. 2021 Jun 11;23(6):e23471. doi: 10.2196/23471 (PMC8386361; doi:10.2196/23471)
Supplement: Multimedia Appendix 2 [file jmir_v23i6e23471_app2.docx]

Imagining the companionship needs of middle-aged and elderly people in their old age
Survey on the Acceptance of Robots vs. Pets and Influencing Factors

Dear Sirs and Ladies:

Thank you sincerely for taking the time to complete this questionnaire!

The purpose of this study is to examine the acceptance of middle-aged and elderly people in Taiwan of **robots** or **pets** as **companions** in their old age and to further analyze the related factors. We hope that the data collected from this questionnaire will provide a better understanding of middle-aged and elderly people's needs for companionship in their old age, and provide a reference and basis for future implementation and policy promotion by the welfare system and care institutions in Taiwan.

This questionnaire is for academic purposes only, and all information is strictly confidential, and no individual information will be disclosed or leaked. Your answers and opinions will have a decisive impact on this study, so please complete the questionnaire according to the actual situation and **do not leave any question out** to ensure the integrity of the questionnaire. If you have any questions, please contact us. Once again, thank you for your cooperation and assistance, and thank you very much!

Institute of Gerontology, National Cheng Kung University

Associate Professor: Ching-Ru Chiu / Graduate Student: Hsieh Hsuan

February, 2018

Part I: Companionship in old age: robots vs. pets

| 1. If you have a robot or a pet as your companion in your old age, how would you feel about it? Please circle one of the following options:  1a. To what extent do I accept a robot as one of my companions in my old age?  I do not accept it at all  I very much look forward to it.  0 1 2 3 4 5 6 7 8 9 10 1b.To what extent do I accept a pet as one of my companions in my old age?  I very much look forward to it.  I do not accept it at all  0 1 2 3 4 5 6 7 8 9 10 |
| --- |
| 2. If you could have a robot to accompany you in your old age, what type or function of robot would you need/prefer?  Please check the following boxes according to your possible preferences and needs (multiple choice): 2a. Service: □①Household service (such as sweeping the floor) □②Health status monitoring □③Safety monitoring  2b. Skills: □①Juggling □②Dancing □③Singing  2c. Interaction: □①Chatting □②Telling stories □③News reporting □④Telling jokes □⑤Talking like a child  2d. Expressions: □①Multiple expression changes  2e. Appearance: □①Animal appearance □②Baby appearance □③Adult appearance □④Non-living appearance  2f.Other: ________________________________________ |
| 3. Do you have any experience in using robots?   (Multiple choice)  □①No experience in using robots  □②Rehabilitation robot, such as assistance in rehabilitation, walking  □③Service robots, such as household assistance (e.g., sweeper robot), health monitoring, safety monitoring  □ ④Companion robot, such as chatting, interaction, storytelling  □ ⑤Other _________________________________________ |
| 4. Have you ever kept a pet in the past?  □①No [Skip to 4c.〕  □②Yes [Continue to answer 4a.〕  4a. How long have you kept your pet?  □①One year or less □②One to five years □③Six to ten years □④Ten years or more  4b. What kind of animals have you kept?  □①Dog □②Cat □③Rabbit □④Fish □⑤Bird □⑥Other ________________  4c. Do you have any experience in animal-assisted therapy?  □①No experience □②Dog doctor □③Equestrian therapy □④Dolphin therapy |

Part II: Physical/Mental State and Activities

5. Five Personalities Scale (IPIP-15) ***Please circle the number from 1 to 5 that best describes your current situation**

|  | Very Not Precise | Somewhat Not Precise | Neutral (Undecided) | Somewhat Precise | Very Precise |
| --- | --- | --- | --- | --- | --- |
| 1. I feel natural when I get along with others | 1 | 2 | 3 | 4 | 5 |
| 2. I initiate conversations with others | 1 | 2 | 3 | 4 | 5 |
| 3. I talk to a lot of different people at parties | 1 | 2 | 3 | 4 | 5 |
| 4. I take time to help others | 1 | 2 | 3 | 4 | 5 |
| 5. I can feel other people's emotions | 1 | 2 | 3 | 4 | 5 |
| 6. I make people feel relaxed when they are with me | 1 | 2 | 3 | 4 | 5 |
| 7. I like to be organized | 1 | 2 | 3 | 4 | 5 |
| 8. I work according to a plan | 1 | 2 | 3 | 4 | 5 |
| 9. I demand accuracy in my work | 1 | 2 | 3 | 4 | 5 |
| 10. I get upset easily | 1 | 2 | 3 | 4 | 5 |
| 11. My mood changes a lot | 1 | 2 | 3 | 4 | 5 |
| 12. I often feel depressed | 1 | 2 | 3 | 4 | 5 |
| 13. I have a rich vocabulary | 1 | 2 | 3 | 4 | 5 |
| 14. I have a vivid imagination | 1 | 2 | 3 | 4 | 5 |
| 15. I always have good ideas | 1 | 2 | 3 | 4 | 5 |

6. Mood Scale

***Everyone has mood changes at times. In the past week, have you had any of the following situations or feelings? Do you have these feelings rarely, sometimes, or often or all the time?**

| **In the past week** | No | Rarely  (only one day) | Sometimes  (two to three days) | Often or always  (four days or more) |
| --- | --- | --- | --- | --- |
| 1. I don't want to eat much because I have a poor appetite | 0 | 1 | 2 | 3 |
| 2. I feel that I am struggling to do everything | 0 | 1 | 2 | 3 |
| 3. I can't sleep well (I can't sleep) | 0 | 1 | 2 | 3 |
| 4. I feel very bad mood | 0 | 1 | 2 | 3 |
| 5.I feel very lonely (lonely, no company) | 0 | 1 | 2 | 3 |
| 6. I think the people around you should not be your companion (not friendly) | 0 | 1 | 2 | 3 |
| 7. I feel sad | 0 | 1 | 2 | 3 |
| 8. I can't work hard to do (I don't have the spirit to do something) | 0 | 1 | 2 | 3 |
| 9. I feel very happy | 0 | 1 | 2 | 3 |
| 10. I think life is going well | 0 | 1 | 2 | 3 |

7. Social participation

***Have you participated in any of the following activities or joined any of these clubs?**

| Type of club or activity | No | Yes |
| --- | --- | --- |
| 1. Community social groups, such as women's clubs, talent classes, etc. | 0 | 1 |
| 2. Religious organizations, such as churches, fellowships, temples, etc. | 0 | 1 |
| 3.Farmers' Association, Fishery Association or Other industrial and commercial organizations of unions, Lions Clubs, etc. | 0 | 1 |
| 4. Political organizations (e.g. political parties, etc.) | 0 | 1 |
| 5. Social service organizations such as Lifeline, Relief Society, Merit Club, volunteer organizations, etc. | 0 | 1 |
| 6. Hometown associations, clansmen associations, etc. | 0 | 1 |
| 7. Senior citizen groups, such as senior citizen associations, Evergreen Clubs, etc. | 0 | 1 |
| 8. Learning activities for the elderly (e.g. tutorial classes, university or academy for the elderly) | 0 | 1 |

8. Leisure activities

***When you are not working, what kind of recreational activities do you do?**

| Types of recreational activities | No | Yes |
| --- | --- | --- |
| 1. Watching TV | 0 | 1 |
| 2. Listening to music or radio | 0 | 1 |
| 3. Reading newspapers, magazines, books or novels, etc. | 0 | 1 |
| 4. Surfing the Internet | 0 | 1 |
| 5. Playing chess or cards (including Mahjong and Four Colour Playing Cards) | 0 | 1 |
| 6. Meeting and chatting with relatives and friends or doing other activities (e.g., making tea for the elderly) | 0 | 1 |
| 7. Meeting and chatting with neighbors or doing other activities (e.g. making tea for the elderly) | 0 | 1 |
| 8. Planting flowers, organizing gardens, gardening, and potted plants (specifically, gardening or potted plants without income) | 0 | 1 |
| 9. Walking | 0 | 1 |
| 10. Bicycle riding | 0 | 1 |
| 11. Jogging, hiking, playing ball and other personal outdoor fitness activities | 0 | 1 |
| 12. Participating in group sports (activities)   (e.g.: singing, dancing, tai chi, external alchemy, karaoke, etc.) | 0 | 1 |

9. Social support

***The following questions are to find out how satisfied you are with the support, comfort and assistance from others (e.g. family, friends, relatives, neighbors, colleagues, etc.) in your daily life during the past six months. In the following questions, please check the following specific boxes according to the actual situation.**

| Social support | Dissatisfied | Neutral (Undecided) | Satisfied |
| --- | --- | --- | --- |
| 1. They listen to you and tell you what's on your mind. | 1 | 2 | 3 |
| 2. They can comfort you when you are in a bad mood. | 1 | 2 | 3 |
| 3. They are there for you when you are in an emergency. | 1 | 2 | 3 |
| 4. They talk to you about interesting things. | 1 | 2 | 3 |
| 5. They spend a good time with you. | 1 | 2 | 3 |
| 6. They spend time with you doing activities that you enjoy. | 1 | 2 | 3 |
| Social Support | Dissatisfied | Neutral (Undecided) | Satisfied |
| 7. They let you know that you can call them for help if you need it. | 1 | 2 | 3 |
| 8. They give you advice or information about your health. | 1 | 2 | 3 |
| 9. They provide you with help with transportation. | 1 | 2 | 3 |
| 10. They lend or give you things you need, other than money. | 1 | 2 | 3 |

Part III: Technology Use and Electronic Literacy

10. Technology and Internet Use

| 1. Do you have any experience using the Internet?  □①No experience □②One year or less □③One to five years □④Six to ten years □⑤Ten years or more |
| --- |
| 2. Do you have any experience in downloading and using mobile apps?  □ ① I have not used Apps at all □ ② I have used Apps but will not download them □ ③ I can download and use Apps by myself |

11. e-health awareness

|  | Strongly disagree | Disagree | Agree | Strongly agree |
| --- | --- | --- | --- | --- |
| 1. I know what health resources are available on the Internet | 1 | 2 | 3 | 4 |
| 2. I know where to find helpful health resources on the Internet | 1 | 2 | 3 | 4 |
| 3. I know how to find helpful health resources on the Internet | 1 | 2 | 3 | 4 |
| 4. I know how to use the Internet to answer health questions | 1 | 2 | 3 | 4 |
| 5. I know how to use health information on the Internet to help myself | 1 | 2 | 3 | 4 |
| 6. I can evaluate health resources on the Internet | 1 | 2 | 3 | 4 |
| 7. I can distinguish the quality of health resources on the Internet | 1 | 2 | 3 | 4 |
| 8.I am confident that I can use information on the Internet to make healthy decisions | 1 | 2 | 3 | 4 |

Part IV: Background Information

| 12. How old are you? ________ |
| --- |
| 13. What is your gender?  □①Male □②Female |
| 14. What is your education level?  □①Elementary school □②Middle school □③High school □④University □⑤Graduate school or above |
| 15. What is your current marital status?  □①unmarried □②married or with a partner □③divorced or separated □④widowed |
| 16. What is your current living situation? Please write down the city you live in ________________  □①city □②countryside |
| 17. What is the type of house you are currently living in?  □①Bungalow □②Townhouse, Total: ________ floors  □③Apartment building without elevator. Total: ________ floors, Living on: ________ floor □④Apartment building with elevator. Total: ________ floors, Living on: ________ floor □⑤Rooftop addition |
| 18. Do you live alone?  □①No □②Yes, I have been living alone for ________ years |
| 19. How many children do you have?  □①No □②Yes, I have ________ children, but I do not live with them □③I have ________ children, and I live with them |
| 20. Do you have a job now?  □①No □②Yes |
| 21. Which of the following categories can you be classified as in your current/pre-retirement occupation?  □①**Type 1 Non-technical workers** Housewife, housekeeper, babysitter, caretaker, temporary worker, cleaner, worker, waiter, vendor, guard, security guard, manager, fisherman, student, unemployed, etc.  □②**Type 2 Semi-skilled workers** Postmen, drivers, shop assistants, shopkeepers, tailors, cooks, salesmen, businessmen, farmers, typists, foremen, supervisors, beauticians, barbers, soldiers, etc.  □③**Type 3 Technical personnel** Bank teller, the ship staff, section members, accountants, cashiers, heads of townships, public opinion representatives, actors, technicians, wholesalers, lieutenant officers, secretaries, scriveners, costume designers, kindergarten teachers, agents, contractors, small business leaders, policemen, firemen, appointed and public officials (technicians, clerks, administrators/executives, staff), etc.  □④**Type 4 Professionals** Elementary and middle school principals, elementary and middle school teachers, accountants, judges, supervisors, attorneys, chiefs of company departments, police officers, school-grade officers, painters, writers, musicians, pharmacists, engineers, architects, managers, managers, assistants, deputies, news or television reporters, county and city councilors, directors of small and medium-sized enterprises, and recommended public officials (technicians, technicians, secretaries)Field officers, painters, writers, musicians, pharmacists, engineers, architects, managers, sub-managers, co-managers, assistant managers, news or TV reporters, county and city councilors, small and medium-sized business leaders, and recommended public officials (engineers, technicians, secretaries, etc.).  □⑤**Type 5 High-level professional management personnel** College presidents, college teachers, physicians, judges, scientists, general officers, legislators, supervisors, examiners, delegates to the National Assembly, presidents, general managers, specially or briefly appointed public officials (ministers, directors, directors), etc. |
| 22. Do you have any of the following diseases?  □①No □②Diabetes □③High blood pressure □④High blood fat □⑤Heart disease □⑥Stroke (cerebrovascular disease)  □⑦ cancer □⑧ chronic liver disease □⑨ kidney disease □⑩ arthritis □ other chronic diseases _____________ |
| 23. What do you think is your current health condition?  □①Very bad □②Bad □③Ordinary □④Good □⑤Very good |
| 24. How do you think your financial situation is?  □①very bad □②bad □③barely enough □④fairly well-off □⑤quite well-off |

**This is the end of the questionnaire. Thank you again for your help！**
